# Retraction: Distinct pathways of ERK1/2 activation by hydroxy-carboxylic acid receptor-1

**DOI:** 10.1371/journal.pone.0320172

**Published:** 2025-03-04

**Authors:** The PLOS One Editors

After this article [1] was published, concerns were raised regarding results presented in Figs 2–6.

Specifically:

- The following lanes appear similar, despite representing different experimental conditions:
  ◦ Fig 2e 3,5-DHBA IB: ERK lanes 2–5 and Fig 4f IB: ERK lanes 1–4
  ◦ Fig 3a IB: ERK lanes 3–5, Fig 3b IB: ERK lanes 1–3 and Fig 6b IB: ERK lanes 4–6 when flipped vertically
  ◦ Fig 3a IB: ERK lanes 1–6 and Fig 6b IB: ERK lanes 3–8 when flipped vertically.
  ◦ Fig 3b IB: ERK lanes 5–10 and Fig 6b IB: ERK lanes 4–9
  ◦ Fig 4b DMSO IB: ERK lanes 1–2 and Fig 4b Go6983 (10 µM) IB: ERK lanes 1–2 when flipped horizontally.
  ◦ Fig 4d IB: ERK lanes 3–7 and Fig 5a DMSO right hand panel lanes 1–5.
- There appear to be vertical discontinuities in the following panels:
  ◦ Fig 6a IB: ERK between lanes 2 and 3.
  ◦ Fig 6c IB: P-ERK between lanes 5 and 6 and between lanes 9 and 10.

The first author stated that errors were made during the preparation of Figs 2–6. The first author provided underlying blot and individual-level quantitative data for some of the panels in Figs 2–6. Upon editorial review, the underlying data provided were insufficient to resolve the above concerns, and raised further concerns for the validity and reliability of the published results in these figures.

In light of the above unresolved concerns, the *PLOS One* Editors retract this article.

GL did not respond to the final editorial decision. HQW, LHW, RPC, and JPL either did not respond directly or could not be reached.
